# Current Practices in Missing Data Handling for Interrupted Time Series Studies Performed on Individual-Level Data: A Scoping Review in Health Research

**DOI:** 10.2147/CLEP.S314020

**Published:** 2021-07-23

**Authors:** Juan Carlos Bazo-Alvarez, Tim P Morris, James R Carpenter, Irene Petersen

**Affiliations:** 1 Research Department of Primary Care and Population Health, University College London (UCL), London, UK; 2 School of Medicine, Universidad Cesar Vallejo, Trujillo, Peru; 3 MRC Clinical Trials Unit at UCL, London, UK; 4 Department of Medical Statistics, London School of Hygiene and Tropical Medicine, London, UK; 5 Department of Clinical Epidemiology, Aarhus University, Aarhus, Denmark

**Keywords:** interrupted time series analysis, segmented regression, missing data, multiple imputation, scoping review

## Abstract

**Objective:**

Missing data can produce biased estimates in interrupted time series (ITS) analyses. We reviewed recent ITS investigations on health topics for determining 1) the data management strategies and statistical analysis performed, 2) how often missing data were considered and, if so, how they were evaluated, reported and handled.

**Study Design and Setting:**

This was a scoping review following standard recommendations from the PRISMA Extension for Scoping Reviews. We included a random sample of all ITS studies that assessed any intervention relevant to health care (eg, policies or programmes) with individual-level data, published in 2019, with abstracts indexed on MEDLINE.

**Results:**

From 732 studies identified, we finally reviewed 60. Reporting of missing data was rare. Data aggregation, statistical tools for modelling population-level data and complete case analyses were preferred, but these can lead to bias when data are missing at random. Seasonality and other time-dependent confounders were rarely accounted for and, when they were, missing data implications were typically ignored. Very few studies reflected on the consequences of missing data.

**Conclusion:**

Handling and reporting of missing data in recent ITS studies performed for health research have many shortcomings compared with best practice.

## Introduction

Interrupted time series (ITS) is a widely used quasi-experimental approach that evaluates the potential impact of an intervention over time, using longitudinal data.^1^ ITS has become more widespread in health research in the past decade.^2^,^3^ The use of observational patient-level data is frequent in ITS,^3–6^ but routinely collected health data usually bring missing data issues.^7^ Hudson et al^3^ detected that only 5% of the ITS studies in health care reported how missing data were handled. Current recommendations in the ITS literature^1^ focus the attention on autocorrelation, seasonality and sample size as potential sources of bias, whereas little advice is given on reporting and handling of missing data.

Missing data management and statistical analysis can be crucial for any ITS study. In a preliminary search,^3–6^ we identified two practices among researchers that could affect the validity of ITS estimates. First, before any statistical analysis, they opt to aggregate individual-level data into population-level data. For example, they average all the available outcome values at each predefined time-point (eg, month) and use these averages as population-level outcome values in the subsequent time series analyses. We call this the “averaging step” and, as we will explain later, this can lead to bias in the “aggregate-level” data analysis. Second, researchers are using statistical tools/approaches for modelling these aggregate-level data (eg, autoregressive integrated moving average [ARIMA]) that have not been designed to account for missing data at the individual level.

ITS guidelines recommend controlling for potential confounders, such as autocorrelation or seasonality, using tools designed for population-level analyses and ignoring the missing data problem at the individual level.^1^,^8–10^ Autocorrelation, whereby two consecutive data points can be more correlated with each other, could appear smaller than if the exact same individuals were included over time. If outcome data from same individuals are missing and not replaced with data from exchangeable individuals, the undervalued autocorrelation can induce bias in the ITS estimates. Seasonality, which is defined by cyclic patterns on the outcome over time at population level, can also be distorted by unseasonal missing data patterns at individual level. Thus, traditional approaches to control for seasonality could be insufficient as well.

Five systematic reviews exploring methodological characteristics of health research with ITS designs have previously been reported.^2^,^3^,^11–13^ They have contributed towards detecting gaps in reporting and the use of standard ITS analyses. However, these studies did not focus on the particular problem of applying these standards to the analysis of individual-level records with missing values. In particular, the missing data issues related to the averaging step and the selected statistical approach have been ignored by previous methodological or review studies of ITS.

Therefore, this review focuses on the practices in missing data handling and analysis that are prevalent in the ITS literature; particularly, how researchers are addressing the problem of having missing data at the individual level.

The present study aims to describe current practices in missing data handling for ITS studies performed in health research. In particular, we were interested in those studies that had access to individual-level data. With this aim, we reviewed ITS investigations on health topics to 1) determine the data management strategies and statistical analyses performed in these studies, and 2) determine how often missing data were considered and, if so, how they were evaluated, reported and handled in the analysis.

## Methods

We conducted this study following the steps previously specified in a scoping review protocol (https://doi.org/10.5522/04/14327717.v1), and following the standard recommendations from the PRISMA Extension for Scoping Reviews.^14^ “Scoping reviews” are alternatives to systematic reviews, which are especially suitable for investigating more general questions such as common practices in research.^15^ For that reason, scoping reviews usually omit any critical appraisal within the reviewed articles, do not assess the risk of bias across the studies and do not end with meta-analyses.

### Inclusion and Exclusion Criteria

We included studies of ITS that assessed any intervention relevant to health care (eg, policies or programmes), with no restrictions on participants, the language of publication or the type of outcome. Both ITS and controlled ITS studies were included. We excluded systematic reviews, meta-analyses, randomised controlled trials, protocols, grey literature (eg, government reports), editorials, letters to editors, retraction papers, methodological studies, studies with no access to individual-level data and studies that used Google Trends data only. Studies whose full text was not available – after trying several avenues – were also excluded. Studies with fewer than two time-points before and two after the first interruption time-point in the ITS were excluded. The access to individual-level data was verified in the methods section of each article, usually in the subsection describing the settings, sample or population studied (eg, if they reported data routinely collected from patients).

### Search Strategy

We used the MEDLINE® and Epub Ahead of Print, In-Process & Other Non-Indexed Citations and Daily database (Ovid version) on 08 February 2020, to identify ITS studies published from 01 January 2019 to 31 December 2019. The search strategy, given in Appendix A, was prepared by the co-authors, and reviewed by an information specialist from the UCL Library.

JCB performed the automatic search with the Ovid tool, removed duplications using EndNote, and screened titles and abstracts manually by the search for inclusion. An independent reviewer (Frank Peralta [FP] orcid.org/0000-0001-5964-6971) double-assessed 10% of same titles and abstracts and, if they agreed, they would proceed to screen using a full-text version of the publications. FP also assessed 10% of the full texts and, in the absence of disagreement, JCB would proceed to randomly select 60 publications for final data extraction, using a random number generator with Stata.^16^ We prespecified a sample size of 60 as enough to obtain information on practices in ITS studies. This decision was based on key questions involving binary answers which could be summarised as proportions. We set our sample size with the following reasoning. The largest standard error (SE) for a proportion with n=60 is for proportion 0.5, which has SE 0.06. For example, estimating that 50% of the studies had adequately handled individual-level missing data would come with 95% CI (37%, 63%). A third colleague was available to help in any disagreement at any stage of this process, whenever it was needed.

### Data Extraction and Analysis

The data extraction form (Appendix B) was reviewed and validated by the co-authors and the information specialist from the UCL Library. From the 60 selected publications, six full-text original articles were randomly selected and were reviewed by FP and JCB independently. If there were no disagreements, JCB would proceed to review the other 54 articles.

Data extracted from the articles can be categorised into three topics: general characteristics, data management and statistical analysis, and missing data reporting and handling.

#### General Characteristics of the ITS Studies

First author, journal, country, study design (eg, ITS, controlled ITS), participants, type of intervention, level of intervention (eg, country, hospital), most granulated cluster available (eg, hospital, individual-level) and longitudinal follow-up (eg, prospective cohort or panel).

#### Data Management and Statistical Analysis

Data source, linked data, outcome type (eg, continuous, proportion), number of time-points, time-point unit, averaging step (yes/no), statistical model (eg, ARIMA, mixed-effects model), confounder reported (yes/no) and confounder adjusted for (yes/no), autocorrelation (considered, tested with, concluded by test, controlled by), seasonality (considered, tested with, concluded by test, controlled by), time-dependent variable (considered, handled by) and other methodological issues (considered, handled by). The averaging step is the step from which the outcome analysed at the population level is the average of more granulated outcome data (eg, individual-level data) at each time point defined for the ITS (eg, one average outcome for each week).

#### Missing Data Reporting and Handling

Missing data considered (yes/no), proportion reported, the missing data mechanism (considered, reported), the method for handling missing data (considered, reported) and sensitivity analysis (considered, reported).

We based this data extraction on the primary outcome, or the first outcome mentioned if the authors did not set a primary outcome.

We summarised data using descriptive statistics (numbers and percentages or median, interquartile range (IQR), minimum and maximum values). Some cross-tabulations of frequencies were used when needed, and these are reported as supplemental material.

## Results

**Figure 1:**
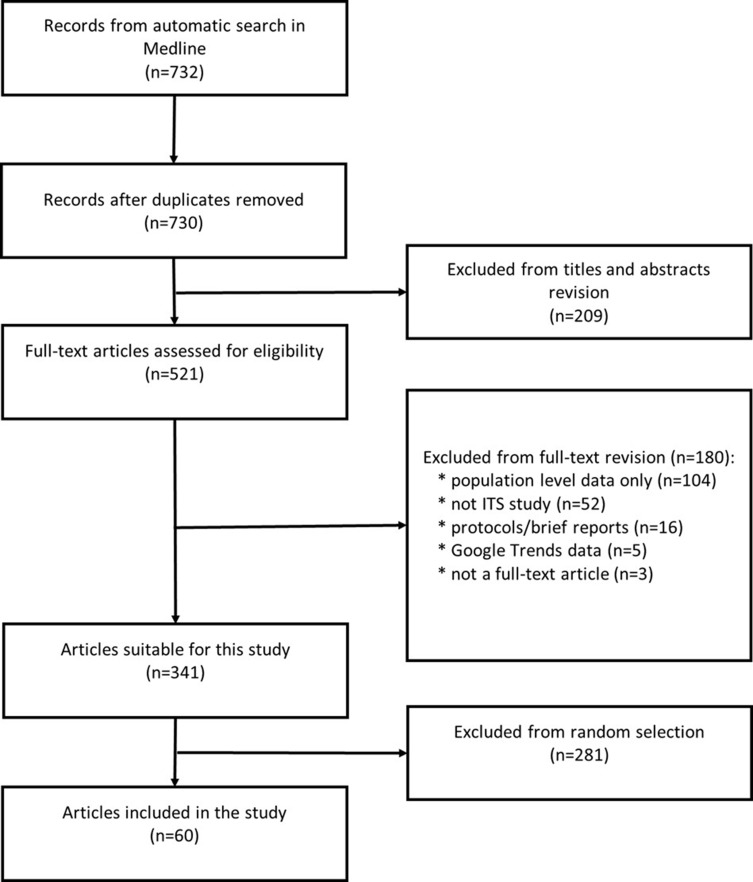
PRISMA diagram for the scoping review. **Notes:** PRISMA figure adapted from Liberati A, Altman D, Tetzlaff J, et al. The PRISMA statement for reporting systematic reviews and meta-analyses of studies that evaluate health care interventions: explanation and elaboration. *Journal of Clinical Epidemiology*. 2009;62(10)e1-e34. Creative Commons.^28^

The search strategy identified 732 titles and abstracts from MEDLINE Ovid (Figure 1). After removing two duplicates, we excluded 209 titles and abstracts that did not meet the inclusion criteria, leaving 521 full-text studies to be checked for eligibility. From this full-text selection, we excluded 180, most of them having only population-level data (n=104). After exclusion, 341 articles were suitable for the final screening; thus, we randomly selected 60 of them, and the list of these studies is provided in Appendix C. Since there were no disagreements during the screening and data extraction process, FP and JCB only double-assessed 10% of the full-text copies.

Most of the 60 studies were from the USA (n=28, 47%), the UK (n=7, 12%) or Canada (n=4, 7%) (Table 1). Only two studies (3%) were not labelled as ITS studies, although they used a single-ITS design (their authors described thems as segmented regression analyses). In total, 48 (80%) studies used a single-ITS design (ie, no control group), whereas 10 (17%) applied a more sophisticated controlled-ITS design.^17^ Patients were the most prevalent participants (n=38, 63%), followed by health personnel (n=6, 10%) or the general population (n=4, 7%). Policies (n=16, 27%), programmes (n=14, 23%) and focused interventions (n=15, 25%) were the most common interventions evaluated. These interventions were frequently applied at a hospital (n=18, 30%) or country level (n=17, 28%). Although all of these studies (n=60, 100%) had access to individual-level data, owing to the nature of the studied ITS outcome (eg, the number of new patients before and after the intervention), only 32 (53%) could have followed/analysed the ITS outcome at an individuallevel (ie, repeated measures of the ITS outcome within individuals). However, other coarser clusters could have been followed over time, for example, hospitals (n=13, 22%), hospital units (n=3, 5%), health facilities (n=3, 5%) or general practices (GPs) (n=3, 5%) (ie, ITS with multiple groups). A cross-table between level of intervention and more granulated clusters available is given in Appendix D. This provides an approximation of how often a researcher could move from modelling population ITS trajectories with population-level data points (ie, only one ITS outcome average at each time-point) to modelling the trajectories with finer level data, which were also available (eg, individual- or hospital-level data for being modelled with mixed-effects or generalised estimating equation [GEE] models). The longitudinal follow-up of the data collected for all of these ITS studies were mostly retrospective and were available at individual level (n=25, 42%) or other less granulated cluster levels (n=21, 35%).

**Table 1:** Characteristics of the Included Interrupted Time Series Studies (N=60)

	n	(%)
**Country of Study**		
Australia	1	(1.7)
Bangladesh	1	(1.7)
Brazil	2	(3.3)
Cambodia	1	(1.7)
Canada	4	(6.7)
China	2	(3.3)
France	2	(3.3)
Germany	1	(1.7)
Israel	1	(1.7)
Italy	1	(1.7)
Japan	1	(1.7)
Malawi	1	(1.7)
Netherlands	1	(1.7)
Rwanda	1	(1.7)
Saudi Arabia	1	(1.7)
South Korea	1	(1.7)
Spain	2	(3.3)
Switzerland	1	(1.7)
UK	7	(11.7)
USA	28	(46.7)
**Study Design**		
CITS	10	(16.7)
ITS	48	(80)
SR	2	(3.3)
**Participants**		
Children	3	(5)
Firefighters	1	(1.7)
General population	4	(6.7)
Health personnel	6	(10)
Health personnel and patients	6	(10)
Insured women	1	(1.7)
Medications	1	(1.7)
Patients	38	(63.3)
**Type of Intervention**		
Guideline/protocol/sound publication or evidence	9	(15)
Focused intervention	15	(25)
Policy	16	(26.7)
Programme	14	(23.3)
Relevant or historical event	3	(5)
Treatment	3	(5)
**Level of Intervention**		
Cities, group of	1	(1.7)
City/district	3	(5)
Country	17	(28.3)
Hospital	18	(30)
Hospitals, group of	8	(13.3)
Individual level	2	(3.3)
State/province/county	10	(16.7)
Fire departments	1	(1.7)
**Most Granulated Cluster Available**		
GP	3	(5)
District	2	(3.3)
Fire department	1	(1.7)
Group of patients (by diagnosis)	1	(1.7)
Health facility	3	(5)
Hospital	13	(21.7)
Hospital unit	3	(5)
Household	1	(1.7)
Individual level	32	(53.3)
Medications	1	(1.7)
**Longitudinal Follow-up**		
Prospective cohort (individuals)	8	(13.3)
Prospective panel (cluster)	6	(10)
Retrospective cohort (individuals)	25	(41.7)
Retrospective panel (cluster)	21	(35)

**Abbreviations:** CITS, controlled interrupted time series; ITS, interrupted time series; SR, segmented regression; GP, general practice.

In most studies, data were routinely collected (n=46, 77%), which is often a source of missing data, since the data collection procedures were not designed for an ITS study or even for any research (eg, ITS outcomes were not collected at similar intervals across patients, such as weight measurement may have been taken at different times) (Table 2). Data were not usually linked to external data (n=10, 17%). The most common ITS outcome type was a proportion (n=39, 65%) and the most common unit of the follow-up time was a month (n=36, 60%). The median number of time-points used in the ITS analysis was 38 (IQR=55). The averaging step was performed in 47 (78%) of the studies. One example of this is illustrated by Close et al.^18^ They averaged the number of stroke admissions per practice per month (73 practices observed from 2011 to 2018), for modelling the ITS on the aggregated data (ie, one average point at each month). They then used an ordinary least squares model with Newey–West standard errors for the ITS analysis. The most typical statistical model was segmented regression (SR) with ordinary least squares estimators (SR-OLS, n=23, 38%) or with maximum likelihood type estimators (SR with generalised linear models [SR-GLM], n=15, 25%). A cross-table between averaging step and statistical model is available in Appendix E, showing how researchers combine them in standard ITS studies. Confounding was considered in 41 (68%) of the studies, but adjustment for confounding was only done in 33 studies (55%).

**Table 2:** Data and Statistical Analyses of the Included Interrupted Time Series Studies (N=60)

	n	(%)
**Data Source**		
Collected for the study (prospective)	14	(23.3)
Routinely collected (retrospective)	46	(76.7)
**Linked Data**		
No	50	(83.3)
Yes	10	(16.7)
**Outcome Type**		
Continuous	10	(16.7)
Count	11	(18.3)
Proportion	39	(65)
**Time-Points, Number**		
Median (IQR)	38	(55)
Minimum	6	
Maximum	1217	
**Time-Points, Unit**		
Day	3	(5)
Half-year	1	(1.7)
Month	36	(60)
Quarter-year	8	(13.3)
Two-month	1	(1.7)
Week	5	(8.3)
Year	6	(10)
**Averaging Step**		
No	11	(18.3)
Yes	47	(78.4)
Unclear	2	(3.3)
**Statistical Model**		
ARIMA	7	(11.7)
Joint-point (exploratory method)	1	(1.7)
SR-GEE	7	(11.6)
SR-GLM	15	(25)
SR-GLS	1	(1.7)
SR-OLS	23	(38.3)
Mixed effects (random intercept only)	4	(6.7)
Mixed effects (random intercept and slopes)	2	(3.3)
**Confounder Reported**		
No	19	(31.7)
Yes	41	(68.3)
**Confounder Adjusted**		
No	27	(45)
Yes	33	(55)

**Abbreviations:** IQR, interquartile range; ARIMA, autoregressive integrated moving average; SR, segmented regression; GEE, generalised estimating equation; GLS, generalised least squares; OLS, ordinary least squares.

Many researchers considered the autocorrelation problem (n=41/60, 68%) (Table 3). However, descriptions about how they tested and handled autocorrelation were sporadic. For example, one-third did not report the test they applied – if any – to evaluate autocorrelation (n=13/41, 32%). This was different for those who worked with individual-level data and fitted GEE or mixed-effects models, reporting within-individual correlation by design (n=11/41, 27%), since they did not usually address the autocorrelation problem at the population level. Among those who identified autocorrelation issues in their data (n=36/41, 88%), the use of Newey–West standard errors (n=7/36, 19%) or autoregressive error terms (n=8/36, 22%) was preferred.

**Table 3:** Reporting and Handling of Methodological Issues in the Included Interrupted Time Series Studies (N=60)

	n	(%)
**Autocorrelation – Considered (n=60)**		
No	19	(31.7)
Yes	41	(68.3)
**Autocorrelation – Tested With (n=41)**		
Breusch–Godfrey	2	(4.9)
Cumby–Huizinga	1	(2.4)
Durbin–Watson	8	(19.5)
Within-individual correlation by design	11	(26.8)
Autocorrelation function	3	(7.3)
Autocorrelation probability	2	(4.9)
Not specified	13	(31.7)
Residuals examination	1	(2.4)
**Autocorrelation – Concluded by Test (n=41)**		
No	5	(12.2)
Yes	36	(87.8)
**Autocorrelation – Controlled By (n=36)**		
Cochrane–Orcutt	1	(2.8)
GEE models	6	(16.7)
Newey–West standard errors	7	(19.4)
Prais–Winsten	2	(5.6)
Autoregressive error term	8	(22.2)
Mixed models	5	(13.9)
Not specified	7	(19.4)
**Seasonality – Considered (n=60)**		
No	41	(68.3)
Yes	19	(31.7)
**Seasonality – Tested With (n=19)**		
Dickey–Fuller	1	(5.3)
Autocorrelation/partial autocorrelation function	2	(10.5)
No formal test	14	(73.7)
Not possible (short period)	1	(5.3)
Regression diagnosis test	1	(5.3)
**Seasonality – Concluded by Test (n=19)**		
No	1	(5.3)
Yes	18	(94.7)
**Seasonality – Controlled By (n=18)**		
ARIMA parameter	1	(5.6)
Covariate in the model	12	(66.7)
Decomposition	1	(5.6)
Not handled (reported as limitation)	2	(11.1)
Seasonal ARIMA	2	(11.1)
**Time-Dependent Variable – Considered (n=60)**		
No	11	(18.3)
Yes	49	(81.7)
**Time-Dependent Variable – Handled By (n=49)**		
Control group	10	(20.4)
Control outcome	1	(2)
Covariate (exploration)	1	(2)
Covariate in the model	3	(6.1)
Reported as a limitation, not handled	34	(69.4)
**Other Issues – Considered (n=60)**		
No	35	(58.3)
Yes	25	(41.7)
**Other Issues – Handled By (n=25)**		
Bonferroni adjustment (*p* values)	1	(4)
Adjusted for survey design	1	(4)
Aggregate ecological design (reported as a limitation)	1	(4)
Confounders not controlled (reported as a limitation)	1	(4)
Minimising immortal time bias	1	(4)
Non-stationary (ARIMA controlled)	2	(8)
Overdispersion evaluation (Poisson models)	2	(8)
Secular trends (reported as a limitation)	2	(8)
Sensitivity analysis (extracting patients)	3	(12)
Sensitivity analysis (impact model)	3	(12)
Sensitivity analysis (various)	6	(24)
Subgroup analysis	2	(8)

**Abbreviations:** GEE, generalised estimating equation; ARIMA, autoregressive integrated moving average.

The seasonality issue was considered in about one-third of the studies (n=19/60, 32%) and, in most cases, it was not formally tested (n=14/19, 74%). In studies with observation periods >1 year (n=52/60, 87%), seasonality was considered in 17/52 (33%). Regardless of the use of a formal test – graphical inspection may have been used, but not described – 18/19 (95%) concluded that there were seasonality effects and made an attempt to control for them. The most popular way to control for seasonality (n=12/18, 67%) was to include covariates of time (eg, dummy variables of months) in the ITS models.

Most studies (n=49/60, 82%) considered that time-dependent confounding could not be handled by a single ITS design.^17^ However, more than two-thirds of the studies only reported the problem as a limitation (n=34/49, 70%), whereas less than one-quarter (n=10/49, 20%) used a control group to address the limitation. Other methodological issues related to the ITS design were also considered (n=25/60, 42%), using sensitivity analyses to evaluate the impact of these issues on the results (n=6/25, 24%). For example, sensitivity analyses were used by extracting groups of patients in order to understand whether unmeasured events – potentially experienced by some groups – could affect the ITS outcome trajectories (n=3/25, 12%). Likewise, sensitivity analyses were applied to contrast the preselected ITS impact model^1^,^19^ with other feasible models (n=3/25, 12%).

Only 13/60 studies (22%) reported issues related to missing data, with considerable variation across the proportion of missing values reported. Although many studies worked on retrospectively collected data (n=46/60, 77%) (Table 2), with irregular recording expected for any outcome at the individual level (n=32/60, 53%) (Table 1), only one study (n=1/13, 8%) explicitly reported this as an issue (missing data on the ITS outcome <60%) (Table 4). Only two studies (n=2/13, 15%) considered the missing data mechanisms and their implications on the analysis, reporting missing at random (MAR) and missing not at random (MNAR) as potential mechanisms behind their missing values (Table 4). The 13 studies reported the method used for handling missing data, complete case analysis (CCA) being the most popular (n=11/13, 85%). Interestingly, in 2/6 investigations using mixed-effects models (Table 2), the researchers recognised how these models can help to handle missing data on the ITS outcome. Missing data were evaluated with sensitivity analyses in two studies only (n=2/13, 15%) (Table 4). In one, results from multiple imputation and CCA were compared. In the other, they compared results from using a missing data indicator – a separate “missing data” – against results from CCA.

**Table 4:** Reporting and Handling of Missing Data Issues in the Included Interrupted Time Series Studies (N=60)

	n	(%)
**Missing Data – Considered (n=60)**		
No	47	(78.3)
Yes	13	(21.7)
**Missing Data – % Reported (n=13)**		
% Not reported, but declared as an issue to be solved	2	(15.4)
Covariates <30%/outcome <50%	1	(7.7)
Covariates at baseline (<1% each, not combined)	1	(7.7)
Covariates at baseline (<10% each, not combined)	2	(15.4)
Covariates at baseline (<2%, flow chart)	1	(7.7)
Covariates at baseline (<25% each, not combined)	1	(7.7)
Covariates at baseline (<25%, flowchart)	1	(7.7)
Covariates at baseline (<30% each, not combined)	1	(7.7)
Covariates at baseline (<5%, flowchart)	1	(7.7)
Outcome <60%	1	(7.7)
Smoking (one case), outcome irregularly recorded	1	(7.7)
**Missing Data Mechanism – Considered (n=13)**		
No	11	(84.6)
Yes	2	(15.4)
**Missing Data Mechanism – Reported (n=2)**		
MAR	1	(50)
MNAR	1	(50)
**Method for Handling Missing Data – Considered (n=13)**		
No	0	(0)
Yes	13	(100)
**Method for Handling Missing Data – Reported (n=13)**		
CCA	11	(84.6)
Mixed intercept model for handling missing outcomes	1	(7.7)
Mixed intercept and slope model for handling missing outcomes	1	(7.7)
**Sensitivity Analysis for Missing Data Mechanism – Considered (n=13)**		
No	11	(84.6)
Yes	2	(15.4)
**Sensitivity Analysis for Missing Data Mechanism – Reported (n=2)**		
Comparing results from MICE versus CCA	1	(50)
Comparing results from using a “missing data category” versus CCA	1	(50)

**Abbreviations:** MAR, missing at random; MNAR, missing not at random; CCA, complete case analysis; MICE, multiple imputation by chained equations.

## Discussion

We identified at least five methodological issues directly associated with missing data handling in ITS studies. First, many studies have been using the averaging step of summarising the ITS outcome at each time-point, even if they had the opportunity to directly model the outcome with longitudinal individual-level data (or at least with data more granulated than the population level in order to avoid data aggregation). Second, analyses of population-level data (eg, aggregate-level SR) were more commonly used than analysis of individual-level data (eg, mixed-effects models). Third, missing data on covariates at baseline are commonly handled by CCA, losing valuable information and potentially leading to bias if ITS estimates are adjusted for these covariates. Fourth, seasonality and other time-dependent confounders are rarely controlled and, when they are, missing data implications are typically ignored. Finally, reporting of missing data was omitted by most ITS studies. Further reflections on the potential consequences of missing data, and the subsequent selection of best methods to handle missing values, are rarely covered in ITS studies.

The averaging step forces the data missing at the individual level to artificially disappear at the population level, generating the false impression of an issue being controlled. For example, if the outcome data are missing at random conditional on a fully observed covariate at the individual level, and we calculate a simple average across individuals at each time-point, the covariate that explains the outcome missingness will become unobserved at the aggregate level (ie, missing not at random; see Appendix F).^20^ This is a potential source of bias that none of the studies reviewed has mentioned as a limitation. The data used in ITS studies are often based on routinely collected data and many such data sources have a large amount of missing outcome data often measured at irregular time intervals. This is particularly important for ITS designs, for which it is expected to have the outcome regularly measured at each time-point.^21^ With many outcome gaps due to irregular recording, researchers frequently selected convenient time periods as units of time (eg, months) and averaged all the available records to set a unique outcome value for each time-point/unit. Many may not be aware that such an approach leads to potential bias owing to data becoming missing not at random, as explained above.

The frequent use of the averaging step seems to be related to the standard use of fixed-effect models, which is abundant in the ITS literature and guidelines.^1^,^3^ Traditionally, ITS analyses have been performed on population-level data and, more recently, on data aggregated to this level.^12^ Researchers would have seen the averaging step as an intuitive way to adapt the individual-level data into the population-level data that guidelines teach them to model.^1^ With no methodological studies on the consequences of the averaging step or recommendations from the ITS guidelines on how to handle missing data,^2^ researchers do not have knowledge of the existence of the issue such that they would be motivated to improve practice.

Most researchers handle missing data on confounders at baseline by using CCA, taking similar actions when modelling interaction terms in controlled ITS studies, but again without major reflection on the implications. For example, if the CCA omitted an observation, the final ITS estimator could reduce precision or be biased.^20^ We have confirmed that the most common approach for handling missing data in the reviewed studies was CCA. Only one study reported the use of multiple imputation and used the standard chained equation method (MICE).^22^ This method may bring congeniality problems when applied to multilevel data (eg, individual follow-up, and adjusting for a dummy month variable at an aggregate level to control for seasonality).^23^ Specification of a congenial imputation model is more complex for MICE when researchers need to introduce time-varying confounders or interaction terms in the models, both of which are expected steps in many controlled ITS studies.^17^ For these more complex scenarios, multilevel multiple imputation methods are preferred^24^ owing to their flexibility to enable interaction terms and confounders to be introduced at different levels.

Seasonality and other time-dependent confounders are barely controlled in ITS studies and, when they are, missing data implications are typically ignored. Following an averaging-step procedure, the missing data at the individual level can affect the way the seasonality is observed at the aggregate level; for example, if the outcome is MAR on sex, while the missingness proportion follows a seasonal pattern for men and data are fully observed for women (see Appendix G). Since the preferred method to control for seasonality seems to be to include a dummy variable of time in the models (eg, month), the control will still incorporate noise from the points describing the seasonality at the aggregate level. Using mixed effects to model the ITS with individual-level data, the seasonality could be controlled by specifying the structure of residual errors (ie, when the intervention is applied at the population level). Under the MAR assumption, these mixed-effect models are unbiased.^25^ However, this or similar control alternatives were not reported by any of the studies included in this review.

There is a lack of missing data reporting in ITS studies, and further reflections on the potential consequences of missing data mechanisms and on the best methods to handle missing values are needed. Previous reviews have found an even lower proportion of missing data reporting,^3^ which indicates that this gap is in the ITS literature. Only one study among all those reviewed reflected on how a potential MNAR mechanism might affect its results.^26^ However, no sensitivity analysis was performed, by any study, to consider the impact that a possible MNAR mechanism could have on the final estimates.^27^ Considering that most of the ITS studies are based on routinely collected data, the control that researchers can have over missing data is minimal; thus, a thorough evaluation/reflection on the missingness mechanisms is the only action that is viable in practice. After such an evaluation, the selection of the best method to handle missing values can be best informed, leading to better alternatives than CCA, which is seldom supported by a rationale in the ITS studies.

In general, the findings of this study are consistent with those reported by previous reviews. From 16 ITS studies published between 1976 and 2011, Polus et al^11^ found five with no statistical models and zero studies using mixed-effect models. Our review focused on 60 publications dated 2019, finding only six studies using mixed-effects models. This combined evidence tells us that researchers’ preferences have not changed dramatically during the past decade. Jandoc et al,^2^ who reviewed ITS studies in drug utilisation published between 1984 and 2013, found that >92% of data sources were administrative data (routinely collected). As they reported a similar proportion of routinely collected data as in this review, we externally confirmed that the missing data prevalence for ITS is still a problem. Hudson et al^3^ reported that continuous ITS outcomes were more frequent, whereas we found that proportion was the most common. Differences seem to come from the way in which Hudson et al would have classified the outcomes. For them, the outcome type would have been defined by the model used (eg, if researchers fitted an OLS model, then the outcome should be typified as continuous). More recently, Turner et al^12^ identified that individual-level outcomes of one type (eg, binary) are often aggregated in population-level outcomes of another type (eg, proportion, counts, rates and continuous), underusing individual-level analysis options (eg, mixed-effects models). Ewusie et al^13^ identified data aggregation in more than one level; for example, average of patients’ data at the hospital level followed by the aggregation of all of the hospitals’ averages into one general average. These findings confirm ours about data aggregation and analysis choices, but also unveil how data manipulation before the statistical analysis occurs in many ways that can often be affected by missing data at the individual level.

We recognise some strengths and limitations in this scoping review. On the strengths side, we followed standard recommendations for performing and reporting scoping reviews.^14^ It is the first time that the missing data handling in ITS studies has been revised and analysed as the main aim of a review. The selected studies come from diverse countries and journals, and the results of this review are consistent with others reported in previous ITS-related reviews,^2^,^3^,^11–13^ which is a good indicator of external validity. Among limitations, we only considered studies which were published in 2019 but, considering outputs from previous ITS-related reviews, it seems unlikely that studies from previous years would have changed the conclusions. We could have missed some publications in the search process owing to the use of just one database (MEDLINE). However, there are no strong reasons to believe that the representativeness of the review has been compromised by this factor. We analysed a random sample from the population of publications (60/341), meaning that our estimates vary about the population value. Thus, although some information about missing data handling in ITS studies could have been omitted, the overall image of the problem studied here is still consistent.

In conclusion, we have demonstrated that reporting and handling of missing data rarely occur in ITS studies performed for health research. Researchers do not tend to evaluate the potential consequences of missing data mechanisms on their ITS estimates; their selection of methods for missing data handling is thus poorly reflected and informed. The complete case analysis is the most commonly applied method, but the control for confounding or interactions can be severely affected by complete case analyses. Data aggregation is also a widespread practice that can affect the validity of the ITS estimates when data are missing at random. To overcome these issues, we recommend that missing data handling should be included ITS guidelines. These guidelines should include a recommendation to explore mixed-effects models and/or multilevel multiple imputation as more efficient analysis alternatives.

## Data Sharing Statement

All papers reviewed in this study are published and available in the biggest databases (some are open access).
